# 1-(4-Chloro­phenyl)-2-[tris­(4-methyl­phenyl)-λ^5^-phosphanyl­idene]butane-1,3-dione

**DOI:** 10.1107/S1600536812051689

**Published:** 2013-01-04

**Authors:** Seyyed Javad Sabounchei, Parisa Shahriary, Faegheh Hosseini Fashami, David Morales-Morales, Simon Hernandez-Ortega

**Affiliations:** a Faculty of Chemistry, Bu-Ali Sina University, Hamedan 65174, Iran; b Instituto de Química, Universidad Nacional Autónoma de México, Circuito Exterior, Ciudad Universitaria, México 04510, Mexico

## Abstract

In the title ylide, C_31_H_28_ClO_2_P [common name α-acetyl-α-*p*-chloro­benzoyl­methyl­enetri(*p*-tol­yl)phospho­rane], the dihedral angle between the 4-chloro­phenyl ring and that of the ylide moiety is 66.15 (10)°. The geometry around the P atom is slightly distorted tetra­hedral [angle range = 105.22 (8)–115.52 (9)°] and the carbonyl O atoms are *syn*-oriented with respect to the P atom. The ylide group is close to planar [maximum deviation from the least-squares plane = 0.006 (2) Å] and the P—C, C—C and C=O bond lengths are consistent with electron delocalization involving the O atoms.

## Related literature

For a general background to organo­phospho­rus compounds and a review of stabilized phospho­nium ylides, see: Bachrach & Nitsche (1994 ▶). For other related literature on ylides, see: Wilson & Tebby (1972 ▶); Sabounchei *et al.* (2010 ▶). For analogous structures, see: Bart (1969 ▶); Kalyanasundari *et al.* (1994 ▶); Sabounchei *et al.* (2007 ▶); Castañeda *et al.* (2001 ▶, 2003 ▶). For bond distance and angle data, see: Dunitz (1979 ▶); Allen *et al.* (1987 ▶).
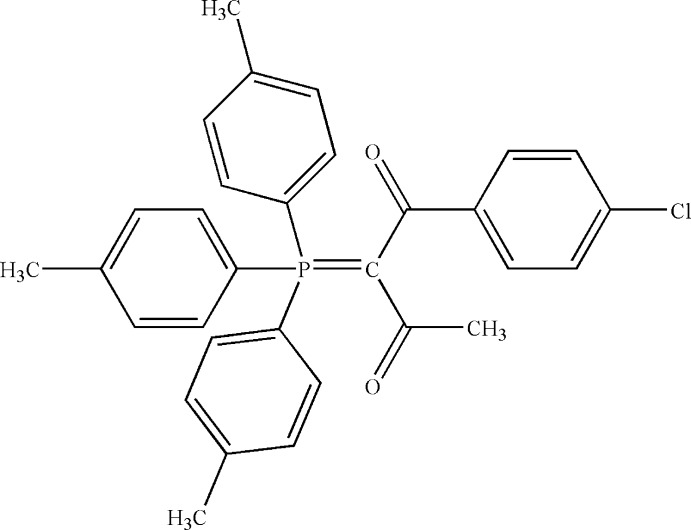


## Experimental

### 

#### Crystal data

C_31_H_28_ClO_2_P
*M* *_r_* = 498.95
Monoclinic, 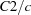

*a* = 20.327 (2) Å
*b* = 14.7560 (15) Å
*c* = 18.9759 (19) Å
β = 113.140 (2)°
*V* = 5233.8 (9) Å^3^
*Z* = 8
Mo *K*α radiation
μ = 0.23 mm^−1^
*T* = 298 K
0.35 × 0.27 × 0.25 mm

#### Data collection

Bruker SMART APEX CCD area-detector diffractometer
21296 measured reflections
4780 independent reflections
3380 reflections with *I* > 2σ(*I*)
*R*_int_ = 0.045

#### Refinement

*R*[*F* ^2^ > 2σ(*F* ^2^)] = 0.040
*wR*(*F* ^2^) = 0.099
*S* = 1.00
4780 reflections
320 parameters
H-atom parameters constrained
Δρ_max_ = 0.23 e Å^−3^
Δρ_min_ = −0.21 e Å^−3^

### 

Data collection: *SMART* (Bruker, 1999 ▶); cell refinement: *SAINT* (Bruker, 1999 ▶); data reduction: *SAINT*; program(s) used to solve structure: *SHELXS97* (Sheldrick, 2008 ▶); program(s) used to refine structure: *SHELXL97* (Sheldrick, 2008 ▶); molecular graphics: *SHELXTL* (Sheldrick, 2008 ▶); software used to prepare material for publication: *SHELXTL*.

## Supplementary Material

Crystal structure: contains datablock(s) I, global. DOI: 10.1107/S1600536812051689/zs2244sup1.cif

Structure factors: contains datablock(s) I. DOI: 10.1107/S1600536812051689/zs2244Isup2.hkl

Supplementary material file. DOI: 10.1107/S1600536812051689/zs2244Isup3.cml

Additional supplementary materials:  crystallographic information; 3D view; checkCIF report

## Figures and Tables

**Fig. 1.**
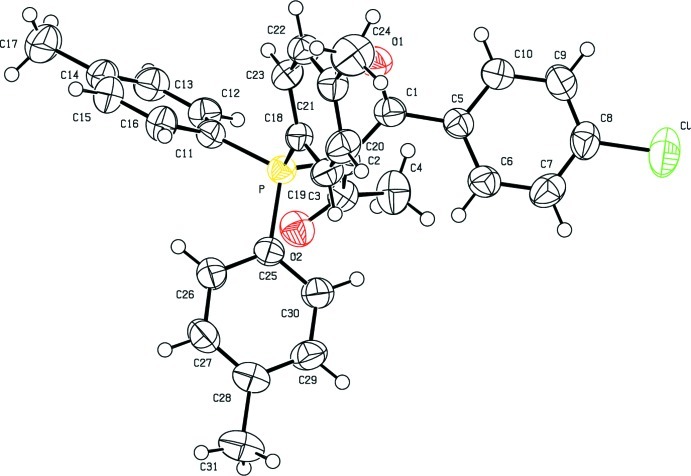
The molecular structure of the title molecule with the atom-numbering scheme. Displacement ellipsoids are drawn at 30% probability level.

## supplementary crystallographic information

### Comment

X-ray structures of stabilized phosphonium ylides possessing a substituent that conjugates with the P═C double bond have been reviewed (Bachrach & Nitsche, 1994). Ylidic resonance is important in phosphonium ylides stabilized by electron-withdrawing substituents due to electronic delocalization between the P—C bond, the ylidic bond, and an acyl group (Castañeda *et al.*, 2001, 2003). In the title compound, C_31_H_28_ClO_2_P (Fig. 1), the dihedral angle between the 4-chlorophenyl ring and the plane of the planar ylide moiety (defined by atoms P, C2, C3, O2, C4) is 66.15 (10)°. The geometry around the P atom is slightly distorted tetrahedral [angle range, 105.22 (8)–115.52 (9)Å]. The P–C2 bond [1.7540 (18) Å] is comparable with analogous distances (Kalyanasundari *et al.*, 1994; Sabounchei *et al.*, 2007) and is longer than the typical P═C double bond in methylenetriphenylphosphorane, Ph_3_P═CH_2_ (Bart, 1969), where there is no opportunity for conjugation with another group. For a similar reason, the C═O bonds are longer than the C═O bonds in ketones (Allen *et al.*, 1987). In the title compound the difference between the C–O bond lengths in the C1–O1–Ph group compared to the C3–O2–CH_3_ group (0.016 Å) may be due to the presence of the extended resonance between the COCH_3_ group and the carbanion. The ylide C-atom is clearly *sp*^2^-hybridized, the sum of the bond angles [359 (4)°] being essentially 360°. The distortions from planarity of the extended ylide group (as induced by non-bonding interactions) are not extreme; the P—C2—C3═O2 torsion angle [2.3 (2)°] suggests a degree of coplanarity and concomitance, but the P—C2—C1—O1 angle [-37.1 (3)°] indicates some rotation of the second carbonyl group out of the plane. In ylides stabilized by a single keto or ester group, there is a strong interaction between cationoid phosphorus and the *syn* acyl O atom (Wilson & Tebby, 1972). The P···O2 [2.853 (1) Å] and P···O1 [3.088 (2) Å] distances are significantly shorter than the sum of the van der Waals radii of P and O (Dunitz, 1979), indicating a strong intramolecular interaction between the P^+^ and O^-^ charge centers, which leads to the *cis* orientation.

### Experimental

A mixture of parachlorobenzoyltri(paratolyl)phosphorane (0.03 mol) and acetic anhydride (0.3 mol) in dry chloroform (10–20 ml) was stirred at 60°C. The reaction was monitored by TLC. The resulting dark solution was evaporated at 80 °C (12 ml) to give a glue which was triturated with ether and the precipitated product was filtered and recrystallized using a solvent diffusion technique (yield; 65%: m.p. 459–458).

### Refinement

The hydrogen atom positions were calculated and refined using a riding model technique, with C—H_aromatic_ = 0.93 Å or C—H_methyl_ = 0.96 Å, with *U*_iso_(H) = 1.2*U*_eq_(C)(aromatic) or 1.5*U*_eq_(C)(methyl).

### Crystal data

**Table:**

C_31_H_28_ClO_2_P	*F*(000) = 2096
*M**_r_* = 498.95	*D*_x_ = 1.266 Mg m^−^^3^
Monoclinic, *C*2/*c*	Melting point = 458–459 K
Hall symbol: -C 2yc	Mo *K*α radiation, λ = 0.71073 Å
*a* = 20.327 (2) Å	Cell parameters from 7723 reflections
*b* = 14.7560 (15) Å	θ = 2.3–25.3°
*c* = 18.9759 (19) Å	µ = 0.23 mm^−^^1^
β = 113.140 (2)°	*T* = 298 K
*V* = 5233.8 (9) Å^3^	Prism, yellow
*Z* = 8	0.35 × 0.27 × 0.25 mm

### Data collection

**Table:**

Bruker SMART APEX CCD area-detector diffractometer	3380 reflections with *I* > 2σ(*I*)
Radiation source: fine-focus sealed tube	*R*_int_ = 0.045
Graphite monochromator	θ_max_ = 25.4°, θ_min_ = 1.8°
Detector resolution: 0.83 pixels mm^-1^	*h* = −24→24
ω scans	*k* = −17→17
21296 measured reflections	*l* = −22→22
4780 independent reflections	

### Refinement

**Table:**

Refinement on *F*^2^	Primary atom site location: structure-invariant direct methods
Least-squares matrix: full	Secondary atom site location: difference Fourier map
*R*[*F*^2^ > 2σ(*F*^2^)] = 0.040	Hydrogen site location: inferred from neighbouring sites
*wR*(*F*^2^) = 0.099	H-atom parameters constrained
*S* = 1.00	*w* = 1/[σ^2^(*F*_o_^2^) + (0.0456*P*)^2^] where *P* = (*F*_o_^2^ + 2*F*_c_^2^)/3
4780 reflections	(Δ/σ)_max_ < 0.001
320 parameters	Δρ_max_ = 0.23 e Å^−^^3^
0 restraints	Δρ_min_ = −0.21 e Å^−^^3^

### Special details

**Table:**

Geometry. All e.s.d.'s (except the e.s.d. in the dihedral angle between two l.s. planes)are estimated using the full covariance matrix. The cell e.s.d.'s are takeninto account individually in the estimation of e.s.d.'s in distances, anglesand torsion angles; correlations between e.s.d.'s in cell parameters are onlyused when they are defined by crystal symmetry. An approximate (isotropic)treatment of cell e.s.d.'s is used for estimating e.s.d.'s involving l.s. planes.
Refinement. Refinement of *F*^2^ against ALL reflections. The weighted *R*-factor *wR* andgoodness of fit *S* are based on *F*^2^, conventional *R*-factors *R* are basedon *F*, with *F* set to zero for negative *F*^2^. The threshold expression of*F*^2^ > σ(*F*^2^) is used only for calculating *R*-factors(gt) *etc*. and isnot relevant to the choice of reflections for refinement. *R*-factors basedon *F*^2^ are statistically about twice as large as those based on *F*, and *R*-factors based on ALL data will be even larger.

### Fractional atomic coordinates and isotropic or equivalent isotropic displacement parameters (Å^2^)

**Table:**

	*x*	*y*	*z*	*U*_iso_*/*U*_eq_	
Cl	0.00240 (4)	0.88102 (4)	0.03610 (4)	0.0896 (3)	
P	0.17486 (3)	0.37797 (3)	0.21740 (3)	0.03935 (15)	
O1	0.16378 (8)	0.56120 (10)	0.29060 (8)	0.0645 (4)	
O2	0.02681 (7)	0.33829 (10)	0.13545 (8)	0.0579 (4)	
C1	0.12109 (10)	0.54772 (13)	0.22438 (11)	0.0454 (5)	
C2	0.10499 (9)	0.45716 (12)	0.19188 (10)	0.0419 (5)	
C3	0.03535 (10)	0.42100 (14)	0.14970 (11)	0.0467 (5)	
C4	−0.03122 (10)	0.47932 (15)	0.12414 (13)	0.0653 (6)	
H4A	−0.0364	0.5109	0.0780	0.098*	
H4B	−0.0271	0.5224	0.1636	0.098*	
H4C	−0.0723	0.4416	0.1146	0.098*	
C5	0.08882 (10)	0.62939 (13)	0.17585 (11)	0.0433 (5)	
C6	0.07281 (10)	0.63087 (14)	0.09771 (12)	0.0507 (5)	
H6	0.0796	0.5788	0.0738	0.061*	
C7	0.04699 (11)	0.70818 (16)	0.05475 (12)	0.0593 (6)	
H7	0.0364	0.7084	0.0024	0.071*	
C8	0.03717 (11)	0.78467 (14)	0.09061 (13)	0.0576 (6)	
C9	0.05338 (12)	0.78600 (15)	0.16758 (13)	0.0644 (6)	
H9	0.0468	0.8385	0.1911	0.077*	
C10	0.07965 (11)	0.70826 (14)	0.21011 (12)	0.0571 (6)	
H10	0.0913	0.7091	0.2627	0.069*	
C11	0.17988 (10)	0.30004 (12)	0.29265 (10)	0.0410 (5)	
C12	0.12272 (11)	0.29172 (13)	0.31441 (11)	0.0492 (5)	
H12	0.0813	0.3251	0.2890	0.059*	
C13	0.12636 (12)	0.23438 (14)	0.37351 (12)	0.0556 (6)	
H13	0.0874	0.2301	0.3874	0.067*	
C14	0.18664 (13)	0.18355 (14)	0.41202 (12)	0.0545 (6)	
C15	0.24374 (12)	0.19226 (14)	0.39040 (12)	0.0570 (6)	
H15	0.2849	0.1584	0.4157	0.068*	
C16	0.24135 (11)	0.24988 (13)	0.33223 (12)	0.0512 (5)	
H16	0.2809	0.2552	0.3194	0.061*	
C17	0.19077 (14)	0.11954 (16)	0.47598 (13)	0.0811 (8)	
H17A	0.1538	0.1343	0.4936	0.122*	
H17B	0.2366	0.1254	0.5176	0.122*	
H17C	0.1845	0.0583	0.4573	0.122*	
C18	0.25965 (9)	0.43610 (12)	0.25043 (10)	0.0389 (4)	
C19	0.28960 (10)	0.46131 (13)	0.19951 (11)	0.0475 (5)	
H19	0.2670	0.4455	0.1481	0.057*	
C20	0.35285 (11)	0.50983 (14)	0.22410 (12)	0.0551 (6)	
H20	0.3723	0.5255	0.1889	0.066*	
C21	0.38777 (10)	0.53554 (13)	0.29975 (12)	0.0497 (5)	
C22	0.35801 (10)	0.50872 (13)	0.35062 (11)	0.0479 (5)	
H22	0.3809	0.5240	0.4021	0.058*	
C23	0.29551 (10)	0.46009 (13)	0.32700 (11)	0.0458 (5)	
H23	0.2769	0.4429	0.3626	0.055*	
C24	0.45494 (12)	0.59149 (17)	0.32627 (14)	0.0790 (8)	
H24A	0.4514	0.6396	0.3586	0.118*	
H24B	0.4611	0.6167	0.2826	0.118*	
H24C	0.4953	0.5539	0.3545	0.118*	
C25	0.16994 (10)	0.31556 (12)	0.13338 (10)	0.0414 (5)	
C26	0.20041 (11)	0.23050 (13)	0.13922 (12)	0.0510 (5)	
H26	0.2223	0.2032	0.1870	0.061*	
C27	0.19849 (12)	0.18588 (14)	0.07444 (13)	0.0571 (6)	
H27	0.2197	0.1291	0.0796	0.069*	
C28	0.16629 (11)	0.22285 (15)	0.00284 (12)	0.0524 (5)	
C29	0.13751 (11)	0.30865 (15)	−0.00266 (11)	0.0587 (6)	
H29	0.1167	0.3362	−0.0505	0.070*	
C30	0.13905 (11)	0.35433 (14)	0.06151 (11)	0.0529 (5)	
H30	0.1190	0.4119	0.0563	0.063*	
C31	0.16111 (13)	0.17156 (16)	−0.06824 (13)	0.0760 (7)	
H31A	0.1908	0.1186	−0.0536	0.114*	
H31B	0.1768	0.2098	−0.0995	0.114*	
H31C	0.1124	0.1538	−0.0967	0.114*	

### Atomic displacement parameters (Å^2^)

**Table:**

	*U*^11^	*U*^22^	*U*^33^	*U*^12^	*U*^13^	*U*^23^
Cl	0.0792 (4)	0.0604 (4)	0.0944 (5)	−0.0098 (3)	−0.0035 (4)	0.0274 (3)
P	0.0384 (3)	0.0401 (3)	0.0382 (3)	0.0009 (2)	0.0137 (2)	−0.0008 (2)
O1	0.0648 (10)	0.0623 (10)	0.0484 (9)	0.0131 (8)	0.0030 (8)	−0.0117 (7)
O2	0.0500 (9)	0.0525 (9)	0.0657 (10)	−0.0059 (7)	0.0169 (7)	−0.0046 (8)
C1	0.0406 (11)	0.0503 (12)	0.0442 (12)	0.0052 (10)	0.0157 (10)	−0.0035 (10)
C2	0.0394 (11)	0.0410 (11)	0.0427 (11)	0.0043 (9)	0.0133 (9)	−0.0001 (9)
C3	0.0446 (12)	0.0486 (13)	0.0450 (12)	0.0018 (10)	0.0156 (10)	0.0029 (10)
C4	0.0407 (12)	0.0631 (14)	0.0856 (17)	0.0021 (11)	0.0179 (12)	0.0124 (13)
C5	0.0392 (11)	0.0422 (11)	0.0464 (12)	−0.0025 (9)	0.0144 (9)	−0.0029 (9)
C6	0.0528 (13)	0.0481 (13)	0.0508 (13)	−0.0071 (10)	0.0199 (11)	−0.0037 (10)
C7	0.0577 (14)	0.0663 (16)	0.0462 (13)	−0.0176 (12)	0.0123 (11)	0.0040 (12)
C8	0.0496 (13)	0.0459 (13)	0.0617 (15)	−0.0097 (10)	0.0051 (11)	0.0094 (11)
C9	0.0725 (16)	0.0437 (13)	0.0666 (16)	0.0046 (12)	0.0162 (13)	−0.0033 (12)
C10	0.0655 (15)	0.0526 (13)	0.0489 (13)	0.0047 (11)	0.0177 (11)	−0.0042 (11)
C11	0.0421 (11)	0.0395 (11)	0.0414 (11)	−0.0013 (9)	0.0163 (9)	−0.0020 (9)
C12	0.0454 (12)	0.0541 (13)	0.0497 (12)	0.0013 (10)	0.0204 (10)	−0.0020 (10)
C13	0.0598 (14)	0.0600 (14)	0.0555 (14)	−0.0097 (12)	0.0317 (12)	−0.0015 (11)
C14	0.0718 (15)	0.0478 (13)	0.0447 (12)	−0.0104 (11)	0.0237 (12)	−0.0004 (10)
C15	0.0588 (14)	0.0524 (13)	0.0566 (14)	0.0074 (11)	0.0191 (12)	0.0109 (11)
C16	0.0465 (12)	0.0535 (13)	0.0555 (13)	0.0044 (10)	0.0221 (11)	0.0072 (10)
C17	0.115 (2)	0.0694 (16)	0.0632 (16)	−0.0135 (15)	0.0399 (16)	0.0115 (13)
C18	0.0393 (11)	0.0383 (11)	0.0376 (11)	0.0040 (8)	0.0136 (9)	0.0014 (8)
C19	0.0509 (12)	0.0535 (12)	0.0371 (11)	−0.0043 (10)	0.0161 (10)	0.0000 (9)
C20	0.0568 (13)	0.0627 (14)	0.0513 (13)	−0.0084 (11)	0.0271 (11)	0.0050 (11)
C21	0.0444 (12)	0.0471 (12)	0.0547 (13)	−0.0018 (10)	0.0163 (11)	0.0025 (10)
C22	0.0424 (12)	0.0524 (13)	0.0428 (12)	−0.0006 (10)	0.0101 (10)	−0.0044 (10)
C23	0.0446 (12)	0.0538 (12)	0.0396 (11)	0.0001 (10)	0.0173 (9)	0.0039 (9)
C24	0.0662 (16)	0.0871 (18)	0.0793 (18)	−0.0277 (14)	0.0238 (14)	−0.0006 (14)
C25	0.0390 (11)	0.0425 (11)	0.0419 (11)	−0.0026 (9)	0.0151 (9)	−0.0032 (9)
C26	0.0624 (14)	0.0430 (12)	0.0512 (13)	0.0019 (10)	0.0263 (11)	0.0014 (10)
C27	0.0722 (15)	0.0414 (12)	0.0666 (15)	0.0004 (11)	0.0367 (13)	−0.0055 (11)
C28	0.0480 (12)	0.0570 (14)	0.0564 (14)	−0.0082 (11)	0.0250 (11)	−0.0144 (11)
C29	0.0563 (14)	0.0719 (16)	0.0402 (12)	0.0089 (12)	0.0106 (10)	−0.0035 (11)
C30	0.0534 (13)	0.0547 (13)	0.0457 (13)	0.0134 (10)	0.0142 (10)	−0.0024 (10)
C31	0.0838 (18)	0.0818 (17)	0.0694 (16)	−0.0112 (15)	0.0376 (14)	−0.0299 (14)

### Geometric parameters (Å, º)

**Table:**

Cl—C8	1.737 (2)	C15—H15	0.9300
P—C2	1.7540 (18)	C16—H16	0.9300
P—C18	1.8028 (18)	C17—H17A	0.9600
P—C11	1.8048 (19)	C17—H17B	0.9600
P—C25	1.8097 (19)	C17—H17C	0.9600
O1—C1	1.231 (2)	C18—C19	1.380 (2)
O2—C3	1.247 (2)	C18—C23	1.391 (2)
C1—C2	1.454 (3)	C19—C20	1.383 (3)
C1—C5	1.502 (3)	C19—H19	0.9300
C2—C3	1.428 (3)	C20—C21	1.381 (3)
C3—C4	1.514 (3)	C20—H20	0.9300
C4—H4A	0.9600	C21—C22	1.383 (3)
C4—H4B	0.9600	C21—C24	1.503 (3)
C4—H4C	0.9600	C22—C23	1.372 (2)
C5—C10	1.381 (3)	C22—H22	0.9300
C5—C6	1.388 (3)	C23—H23	0.9300
C6—C7	1.380 (3)	C24—H24A	0.9600
C6—H6	0.9300	C24—H24B	0.9600
C7—C8	1.373 (3)	C24—H24C	0.9600
C7—H7	0.9300	C25—C30	1.381 (3)
C8—C9	1.365 (3)	C25—C26	1.385 (3)
C9—C10	1.383 (3)	C26—C27	1.382 (3)
C9—H9	0.9300	C26—H26	0.9300
C10—H10	0.9300	C27—C28	1.368 (3)
C11—C12	1.383 (2)	C27—H27	0.9300
C11—C16	1.391 (3)	C28—C29	1.381 (3)
C12—C13	1.383 (3)	C28—C31	1.514 (3)
C12—H12	0.9300	C29—C30	1.381 (3)
C13—C14	1.376 (3)	C29—H29	0.9300
C13—H13	0.9300	C30—H30	0.9300
C14—C15	1.380 (3)	C31—H31A	0.9600
C14—C17	1.514 (3)	C31—H31B	0.9600
C15—C16	1.379 (3)	C31—H31C	0.9600
			
C2—P—C18	109.80 (9)	C11—C16—H16	119.9
C2—P—C11	115.52 (9)	C14—C17—H17A	109.5
C18—P—C11	106.20 (8)	C14—C17—H17B	109.5
C2—P—C25	109.72 (9)	H17A—C17—H17B	109.5
C18—P—C25	105.22 (8)	C14—C17—H17C	109.5
C11—P—C25	109.83 (9)	H17A—C17—H17C	109.5
O1—C1—C2	122.17 (18)	H17B—C17—H17C	109.5
O1—C1—C5	117.33 (17)	C19—C18—C23	117.93 (17)
C2—C1—C5	120.40 (17)	C19—C18—P	120.62 (14)
C3—C2—C1	126.13 (17)	C23—C18—P	121.40 (14)
C3—C2—P	114.99 (14)	C18—C19—C20	120.71 (18)
C1—C2—P	118.18 (14)	C18—C19—H19	119.6
O2—C3—C2	120.67 (18)	C20—C19—H19	119.6
O2—C3—C4	117.14 (18)	C21—C20—C19	121.54 (18)
C2—C3—C4	122.11 (18)	C21—C20—H20	119.2
C3—C4—H4A	109.5	C19—C20—H20	119.2
C3—C4—H4B	109.5	C20—C21—C22	117.36 (18)
H4A—C4—H4B	109.5	C20—C21—C24	121.81 (19)
C3—C4—H4C	109.5	C22—C21—C24	120.83 (19)
H4A—C4—H4C	109.5	C23—C22—C21	121.64 (18)
H4B—C4—H4C	109.5	C23—C22—H22	119.2
C10—C5—C6	118.13 (18)	C21—C22—H22	119.2
C10—C5—C1	119.65 (18)	C22—C23—C18	120.80 (18)
C6—C5—C1	122.03 (17)	C22—C23—H23	119.6
C7—C6—C5	121.3 (2)	C18—C23—H23	119.6
C7—C6—H6	119.4	C21—C24—H24A	109.5
C5—C6—H6	119.4	C21—C24—H24B	109.5
C8—C7—C6	118.8 (2)	H24A—C24—H24B	109.5
C8—C7—H7	120.6	C21—C24—H24C	109.5
C6—C7—H7	120.6	H24A—C24—H24C	109.5
C9—C8—C7	121.4 (2)	H24B—C24—H24C	109.5
C9—C8—Cl	119.74 (19)	C30—C25—C26	118.02 (18)
C7—C8—Cl	118.84 (18)	C30—C25—P	120.35 (15)
C8—C9—C10	119.2 (2)	C26—C25—P	121.51 (15)
C8—C9—H9	120.4	C27—C26—C25	120.36 (19)
C10—C9—H9	120.4	C27—C26—H26	119.8
C5—C10—C9	121.1 (2)	C25—C26—H26	119.8
C5—C10—H10	119.4	C28—C27—C26	121.9 (2)
C9—C10—H10	119.4	C28—C27—H27	119.0
C12—C11—C16	118.25 (18)	C26—C27—H27	119.0
C12—C11—P	119.99 (15)	C27—C28—C29	117.62 (19)
C16—C11—P	121.71 (15)	C27—C28—C31	121.6 (2)
C11—C12—C13	120.85 (19)	C29—C28—C31	120.8 (2)
C11—C12—H12	119.6	C30—C29—C28	121.2 (2)
C13—C12—H12	119.6	C30—C29—H29	119.4
C14—C13—C12	121.1 (2)	C28—C29—H29	119.4
C14—C13—H13	119.5	C29—C30—C25	120.82 (19)
C12—C13—H13	119.5	C29—C30—H30	119.6
C13—C14—C15	117.94 (19)	C25—C30—H30	119.6
C13—C14—C17	121.5 (2)	C28—C31—H31A	109.5
C15—C14—C17	120.5 (2)	C28—C31—H31B	109.5
C16—C15—C14	121.7 (2)	H31A—C31—H31B	109.5
C16—C15—H15	119.1	C28—C31—H31C	109.5
C14—C15—H15	119.1	H31A—C31—H31C	109.5
C15—C16—C11	120.1 (2)	H31B—C31—H31C	109.5
C15—C16—H16	119.9		
			
O1—C1—C2—C3	132.8 (2)	C12—C13—C14—C17	−179.07 (19)
C5—C1—C2—C3	−50.8 (3)	C13—C14—C15—C16	0.1 (3)
O1—C1—C2—P	−37.1 (3)	C17—C14—C15—C16	179.9 (2)
C5—C1—C2—P	139.25 (15)	C14—C15—C16—C11	−1.1 (3)
C18—P—C2—C3	167.12 (14)	C12—C11—C16—C15	1.4 (3)
C11—P—C2—C3	−72.84 (16)	P—C11—C16—C15	178.76 (15)
C25—P—C2—C3	51.94 (17)	C2—P—C18—C19	−89.06 (16)
C18—P—C2—C1	−21.85 (17)	C11—P—C18—C19	145.38 (15)
C11—P—C2—C1	98.18 (16)	C25—P—C18—C19	28.95 (17)
C25—P—C2—C1	−137.04 (15)	C2—P—C18—C23	88.39 (16)
C1—C2—C3—O2	−167.91 (18)	C11—P—C18—C23	−37.16 (17)
P—C2—C3—O2	2.3 (2)	C25—P—C18—C23	−153.59 (15)
C1—C2—C3—C4	8.8 (3)	C23—C18—C19—C20	−0.8 (3)
P—C2—C3—C4	178.97 (15)	P—C18—C19—C20	176.78 (15)
O1—C1—C5—C10	−29.4 (3)	C18—C19—C20—C21	−0.7 (3)
C2—C1—C5—C10	154.09 (18)	C19—C20—C21—C22	1.7 (3)
O1—C1—C5—C6	145.43 (19)	C19—C20—C21—C24	−177.5 (2)
C2—C1—C5—C6	−31.1 (3)	C20—C21—C22—C23	−1.3 (3)
C10—C5—C6—C7	−1.3 (3)	C24—C21—C22—C23	177.91 (19)
C1—C5—C6—C7	−176.25 (18)	C21—C22—C23—C18	−0.1 (3)
C5—C6—C7—C8	0.0 (3)	C19—C18—C23—C22	1.2 (3)
C6—C7—C8—C9	1.0 (3)	P—C18—C23—C22	−176.35 (14)
C6—C7—C8—Cl	−178.27 (15)	C2—P—C25—C30	27.03 (18)
C7—C8—C9—C10	−0.6 (3)	C18—P—C25—C30	−91.03 (17)
Cl—C8—C9—C10	178.66 (17)	C11—P—C25—C30	155.04 (15)
C6—C5—C10—C9	1.7 (3)	C2—P—C25—C26	−156.82 (16)
C1—C5—C10—C9	176.79 (19)	C18—P—C25—C26	85.11 (17)
C8—C9—C10—C5	−0.8 (3)	C11—P—C25—C26	−28.82 (18)
C2—P—C11—C12	14.15 (18)	C30—C25—C26—C27	−1.1 (3)
C18—P—C11—C12	136.13 (15)	P—C25—C26—C27	−177.29 (16)
C25—P—C11—C12	−110.57 (16)	C25—C26—C27—C28	−0.7 (3)
C2—P—C11—C16	−163.22 (15)	C26—C27—C28—C29	2.2 (3)
C18—P—C11—C16	−41.24 (18)	C26—C27—C28—C31	−176.8 (2)
C25—P—C11—C16	72.06 (18)	C27—C28—C29—C30	−2.1 (3)
C16—C11—C12—C13	−0.6 (3)	C31—C28—C29—C30	177.0 (2)
P—C11—C12—C13	−178.05 (15)	C28—C29—C30—C25	0.4 (3)
C11—C12—C13—C14	−0.4 (3)	C26—C25—C30—C29	1.2 (3)
C12—C13—C14—C15	0.7 (3)	P—C25—C30—C29	177.46 (16)
